# Supplementary material for: A Histological and Morphometric Assessment of the Adult and Juvenile Rat Livers after Mild Traumatic Brain Injury
Source: Cells. 2021 May 6;10(5):1121. doi: 10.3390/cells10051121 (PMC8148537; doi:10.3390/cells10051121)
Supplement: Supplementary file 1 [file cells-10-01121-s001.zip › cells-1161852-supplementary.pdf]

cells

MDPI

Supplementary Materials

A histological and morphometric assessment of the adult and juvenile rat livers after mild traumatic brain injury

Ruslan Prus, Olena Appelhans, Maksim Logash, Petro Pokotylo, Grzegorz Józef Nowicki and Barbara Ślusarska

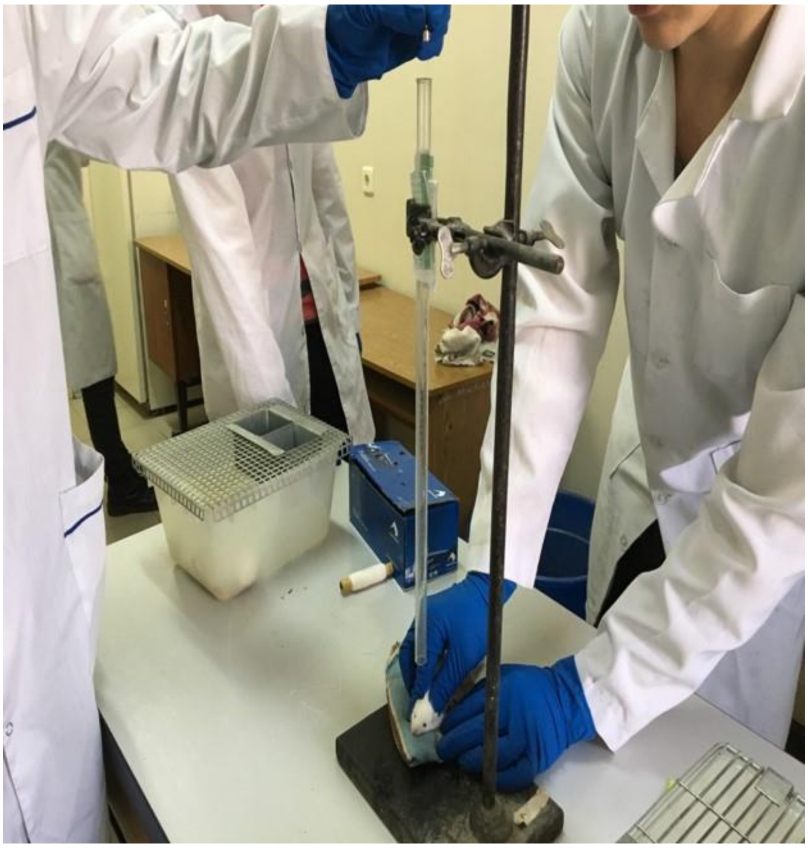

Figure S1. Photographic representation of the platform used in the experiment to induce mTBI in tested rats [23].

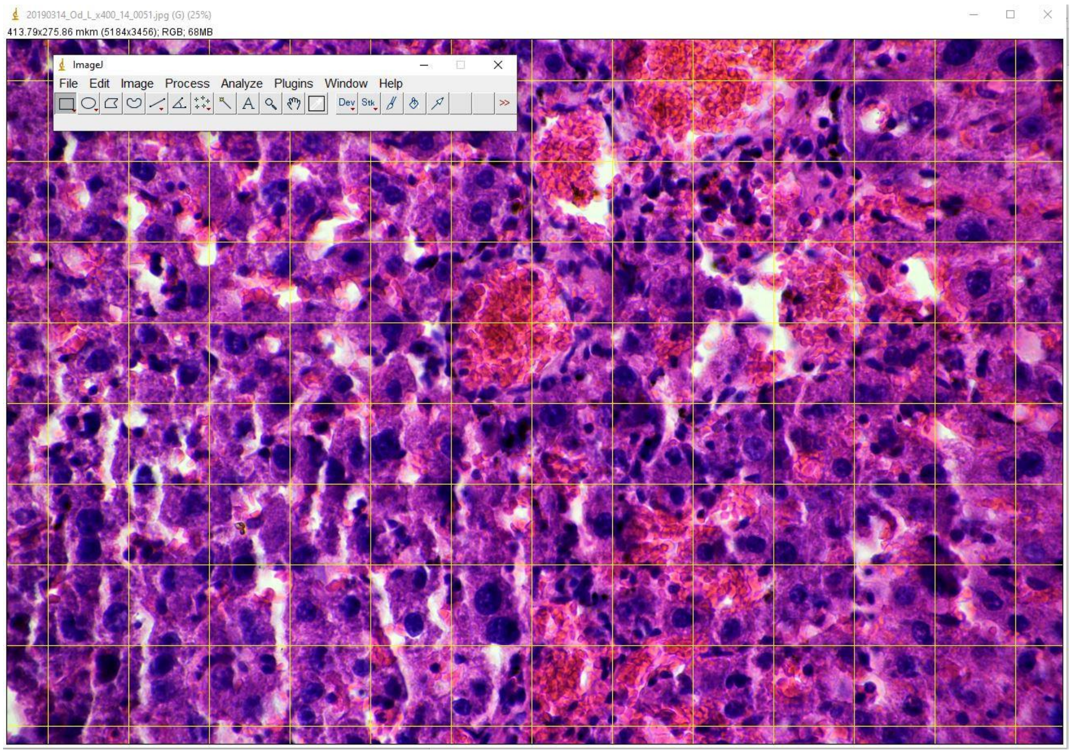

Figure S2. ImageJ software with a "Grid" tool over the image.

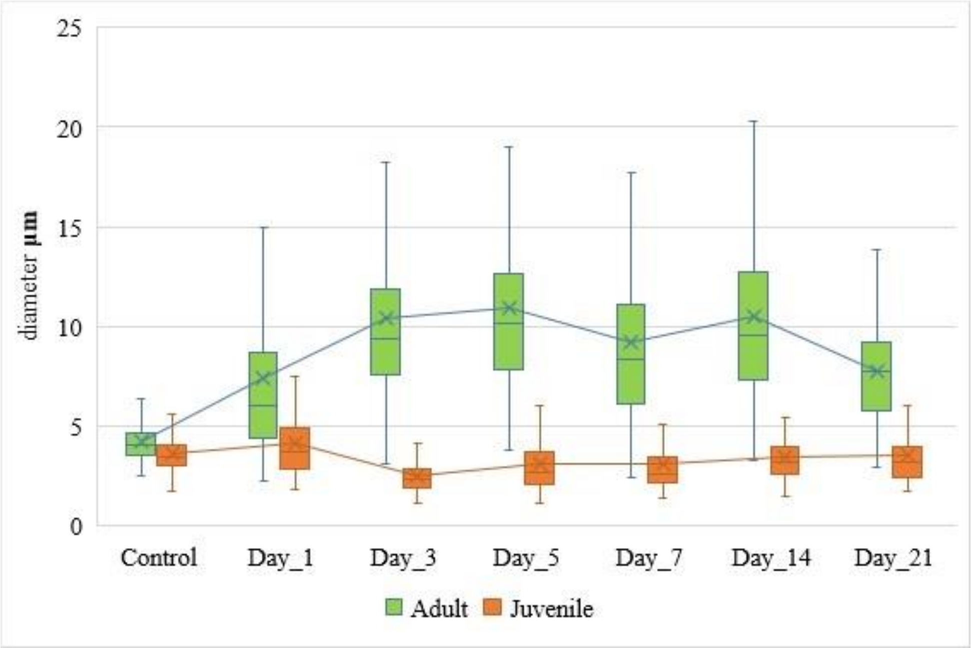

Figure S3. Diameter of rat's liver sinusoids (boxplots of data without outliers) of adult and juvenile rats after mTBI.
